# The Cerebro-Morphological Fingerprint of a Progeroid Syndrome: White Matter Changes Correlate with Neurological Symptoms in Xeroderma Pigmentosum

**DOI:** 10.1371/journal.pone.0030926

**Published:** 2012-02-21

**Authors:** Jan Kassubek, Anne-Dorte Sperfeld, Elmar H. Pinkhardt, Alexander Unrath, Hans-Peter Müller, Karin Scharffetter-Kochanek, Albert C. Ludolph, Mark Berneburg

**Affiliations:** 1 Department of Neurology, University of Ulm, Ulm, Germany; 2 Department of Dermatology, University of Ulm, Ulm, Germany; 3 Department of Dermatology, Eberhard Karls University Tübingen, Tübingen, Germany; Beijing Normal University, Beijing, China

## Abstract

**Background:**

Xeroderma pigmentosum (XP) is a rare autosomal recessive progeroid syndrome. It has recently been shown that the underlying DNA repair defect plays a central role in the aging process. In addition to skin symptoms, various premature neurological abnormalities have been reported.

**Methodology/Principal Findings:**

We present the clinical neurological phenotype in 14 XP patients (seven subtypes), in seven of these patients together with conventional and multiparametric advanced MRI data to assess the macrostructural and microstructural cerebral morphology in comparison to controls, including volumetric measurements, MR spectroscopy (^1^H MRS), and diffusion tensor imaging (DTI). Clinical hallmarks were spinocerebellar ataxia, pyramidal tract signs, and mild cognitive deficits. DTI demonstrated significantly reduced WM directionality in all regions investigated, i.e. the thalamus, the corticospinal tracts and the dorsal corpus callosum. Single patients showed a marked relative hippocampal volume reduction, but the patients were not different from controls in the volumetric measurements of hippocampal and whole brain volumes at group level. However, ^1^H MRS demonstrated that the hippocampal formation was metabolically altered.

**Conclusions:**

The most prominent feature was the white matter affectation, as assessed by DTI, with volume and directionality reductions of the fiber projections involving both the craniocaudal fibers and the interhemispheric connections. These findings, although heterogeneous among the study sample, could be correlated with the clinico-neurological symptoms. The imaging findings support the position that myelin structures degrade prematurely in the brain of XP patients.

## Introduction

Nucleotide excision repair (NER) is a mechanism removing bulky, helix distorting DNA lesions [1]. Defects in NER have been shown to play a role in cancer as well as aging, and recent publications indicate that NER deficiencies lead to premature aging through suppression of the somatotroph axis [2]. Deficiencies of NER lead to the progeroid syndrome xeroderma pigmentosum (XP) [3]. XP is an autosomal recessive disease with an incidence of 1 patient per 10^6^ livebirths [4]. Clinically, it is characterised by premature development of sun-sensitive, xerotic skin, hypo- and hyperpigmentation, telangiectasia and a highly increased skin cancer risk [5], [6].

There are seven different subgroups of XP plus a variant. The seven complementation groups are named according to the correspondingly defective NER-protein (XP A–G) and the variant shows a defect in the translesion-DNA polymerase eta. In addition to dermatological symptoms, XP patients also show premature development of neurological abnormalities. The first detailed description of XP in combination with neurological abnormalities dated from 1932 [7]. In the late 1960s, XP was described as the first human neurodegenerative disease caused by inherited defects in DNA repair mechanisms [8], [9], [10]. Most of the neurological abnormalities were found in the XP-A complementation group – the most common form in Japan – and to a lesser extent in XP-D and XP-G. Reported neurological signs and symptoms were progressive and included peripheral neuropathy, deafness, bulbar, extrapyramidal, and cerebellar symptoms, corticospinal tract abnormalities, and mental deterioration [5], [10], [11], [12]. These XP-A, XP-D, and XP-G patients established the existence of the ‘XP neurological disease’ as a distinct clinical entity. To date, it is accepted that up to 30% of all XP patients present with neurological abnormalities [5], [13], [14] although the phenotype remains ill-defined and it is unclear precisely which factor determines severity and onset of neurological symptoms. Childhood and early adult manifestations are known [10], and XP with childhood onset is often associated with intellectual impairment at different degrees and a delayed motor development. Thus, a mixture of developmental aberrations and degenerative features could be observed. In a recent longitudinal investigation in 16 Finnish XP patients, a spectrum of neurological signs and symptoms was reported [15]. Based on this and the fact that molecular defects in XP apparently influences mitotic and postmitotic tissue, a detailed brain imaging evaluation of patients with various XP forms with respect to the neurological presentation seems to be essential. In the present study, we describe the clinical neurological phenotype in a cohort of German XP patients as confirmed by measurement of reduced DNA repair (unscheduled DNA synthesis (UDS)). It has previously been published that XP patients with neurological symptoms show mostly white matter pathology but also gray matter pathology in the hippocampus and striatum [15], but numbers were small in the tested cohort. In order to identify the cerebral morphological correlates, we investigated their brain structures by use of advanced MRI including multi-parametric MRI applications such as volumetry, proton MR spectroscopy (^1^H MRS), and diffusion tensor imaging (DTI). With respect to the a priori hypotheses, we acquired ^1^H MRS data in the hippocampus and performed hippocampal volumetry since we considered the hippocampus the most promising region-of-interest (ROI) according to the above-named previous report. For the probably more widespread white matter alterations, we used an explorative approach by an initial whole brain-based analysis to define the ROIs for further analysis.

## Materials and Methods

### 2.1. Patients

Patients were recruited from the outpatient clinic of the Department of Dermatology, University of Tübingen, Germany. All study investigations were performed in the Department of Neurology, University of Ulm. Patients or their caregivers/parents gave informed consent to participate in the study which had been approved by the local Ethics Committees of the Universities of Ulm and Tübingen.

A detailed general and neurological examination was carried out. Skin changes observed in the study patients did not differ from the spectrum of dermatological symptoms previously published for XP including photosensitivity, xerosis cutis, poikiloderma, telangiectasia, squamous and basal cell carcinoma as well as malignant melanoma. Dermatological features will thus not be detailed in the following.

The patient's descriptions are focused on medical history and symptoms related to potential nervous system abnormalities. In case of clinical evidence for a peripheral neuropathy and the patient's consent to participate, it was confirmed by a neurophysiological investigation according to clinical standards including the measurement of the nerve conduction velocity (NCV) and electromyography (Multiliner® Evolution 1.63; Jaeger/Toennies, Hoechberg, Germany).

### 2.2 Neuroimaging: MRI data acquisition and postprocessing

The same MRI protocol was acquired in all patients using the same 1.5 Tesla MR system (Magnetom Symphony®, Siemens, Erlangen, Germany), equipped with a standard headcoil. Conventional MRI included routine sequences of T1 and T2-weighted imaging; for a synopsis of the sequences and parameters cf. **Table 1**. No gap was used in the acquisition of MRI slices. In order to avoid movement artefacts during acquisition, a vacuum mold adapted to the subjects' heads was used, and the young patients were calmed by presence of one of their parents in the scanning room. Post acquisition, all data sets were thoroughly checked for motion artefacts, and only those MRI data without any motion artefacts were used.

**Table 1:** MRI protocol: both conventional MRI sequences (columns 1–4) and non-routine sequences (columns 5–6) are included.

sequence parameters	T1w SE	T2w TSE	T2w FLAIR	DWI	MP-RAGE	DTI
repetition time (TR) (ms)	456	3760	9000	3400	1740	8000
time to echo (TE) (ms)	12	98	130	97	3.42	93
inversion time (TI) (ms)	–	–	2400	–	880	–
field of view (FOV) (mm)	230	230	230	230	250	192
matrix	192×256	358×512	202×256	128×128	256×256	128×128
orientation	sagittal	axial	coronar	axial	sagittal	axial
No of slices (n)	19	19	19	19	192 (3D)	45
slice thickness (mm)	5	5	5	5	1	2.2

T1/T2 w, T1/T2 weighted; SE, spin echo; TSE, turbo spin echo; FLAIR, fluid-attenuated inversion recovery; DWI, diffusion weighted imaging; MP-RAGE, magnetization prepared rapid acquisition gradient echo; DTI, diffusion tensor imaging. Note that ^1^H MRS acquisition parameters are listed separately in the text.

In seven patients, MP-RAGE data for volumetry and ^1^H MRS data with hippocampal volumes of interest (VOIs) were acquired (i.e., patients No. 1, 3, 4, 8, 12, 13, 14). For the demographic data and the clinical neurological presentation of these patients, please refer to Tables 2 and 3. The remaining patients could not be investigated by neuroimaging due to fear and restlessness. In six of the patients who received MRI, an additional cerebellar VOI could be investigated by ^1^H MRS (exception, case 12). DTI data were acquired in five patients (patients No. 4, 8, 12, 13, 14). In order to compare the data of all MRI studies to a normal data base, a sample of seven healthy controls were investigated who were pairwise age-matched (maximum difference, ±4 years) and gender-matched. (In the only juvenile patient of the MRI group, i.e. patient 3 aged 12 years, the matched control was also exactly 12 years old.) The control subjects received the identical MRI scanning protocol as the patients.

**Table 2:** Complementation group, sex, nutritional status and ages at XP- or neurological symptom onset and at recent investigation are given.

	1	2	3	4	5	6	7	8	9	10	11	12	13	14
XP complementation group	A	A	C	C	C	C	C	C	D	D	XP-D/TTD	F	V	na
age at XP onset [years]	<4	1	<1	<1	7	1.5	1	2	<2	1	<2	10	21	5
age at neurological manifestation [years]	23	<3	2	-	<1	-	3	20	5	-	<2	68	-	5
age at investigation [years]	23	16	12	23	7	36	5	20	5	27	10	68	38	23
sex	m	m	m	f	f	f	m	m	m	m	f	f	m	f
BMI [kg/m^2^]	26.2	unk	18.2	20.1	unk	23.6	15.4	23.1	unk	unk	12.9	18.7	30.2	unk

TTD, Trichothiodystrophy; na, not assigned; unk, unknown.

**Table 3:** Neurological and habit conditions in the XP patients compared to the knowledge from literature.

			literature											Present study										
	XP Complementation group	A	B	C	D	E	F	G	V	TTD	A	A	C	C	C	C	C	C	D	D	D/TTD	F	V	na
	Case number										1	2	3	4	5	6	7	8	9	10	11	12	13	14
Neurological abnormalities	Mental retardation	+	−	+	+	−	+	+	−	+	−	+	−	−	−	−	−	+	−	−	+	−	−	+
	abnormal behaviour	−	−	(+)	−	−	−	−	−	+	−	+	−	−	+	−	+	−	+	−	−	−	−	+
	dementia	−	−	−	−	−	−	+	−	−	−	−	−	−	−	−	−	−	−	−	−	−	−	+
	Reduced psychomotoric development	+	−	−	−	−	−	+	−	+	−	+	−	−	+	−	−	−	−	−	+	−	−	+
	Gait disturbances	+	−	+	−	−	+	+	−	+	−	+	−	−	+	−	−	+	−	−	+	−	−	+
	ataxia	+	+	+	+	−	+	+	−	+	−	+	(+)	−	+	−	+	+	+	−	+	+	−	+
	Cerebellar dysfunction	+	+	−	−	−	−	−	−	+	+	+	−	−	−	−	−	−	−	−	+	−	−	+
	Pyramidal tract signs	+	+	−	+	−	−	+	−	+	−	−	+	−	−	−	−	+	−	−	+	−	−	+
	Hyporeflexia	+	−	(+)	+	−	−	−	−	+	(+)*	−	−	−	−	−	−	−	−	−	+	+	−	−
	Sensory deficits	+	−	−	−	−	−	−	−	−	−	−	−	−	−	−	−	−	−	−	−	+	−	−
	dysarthria	+	−	(+)	−	−	−	−	−	+	−	+	−	−	−	−	−	+	−	−	+	−	−	+
	myoclonus	−	−	−	−	−	−	−	−	−	−	−	−	−	−	−	−	−	−	−	+	−	−	+
	Choreoathetosis	+	−	−	+	−	−	−	−	+	−	−	−	−	−	−	−	−	−	−	−	−	−	−
	tremor	−	−	−	−	−	−	−	−	−	−	−	−	−	−	−	−	−	−	−	+	−	−	−
	seizures	+	−	−	−	−	−	−	−	+	−	−	−	−	−	−	−	−	−	−	−	−	−	+
	Peripheral nerves																							
	Reduced NCV	+	−	−	+	−	−	+	−	+	−	−	−	−	−	−	−	−	−	−	+	(+)	−	−
	Axonal Sensorimotor neuropathy	+	−	−	−	−	−	+	−	−	−	−	−	−	−	−	−	−	−	−	+	+	−	−
	Isolated LMND	−	−	+	+	−	−	−	−	−	−	−	−	−	−	−	−	−	−	−	−	−	−	−
	Sensorineural deafness	+	+	+	+	−	+	+	−	+	−	−	−	−	−	−	−	−	−	−	−	−	−	−
	Autonomic dysfunction	+	−	−	−	−	+	−	−	−	−	−	−	−	−	−	−	−	−	−	−	−	−	−
																								
Habit	Short stature, dwarfism	+	−	−	+	−	+	+	−	+	−	+	−	+	−	−	−	+	−	−	+	−	−	+
	skeletal dysplasia	−	−	−	−	−	+	−	−	+	−	−	−	−	−	−	−	−	−	−	+	−	−	+
	Progeroid appearance	−	−	−	+	−	+	+	−	+	−	−	−	−	−	−	−	−	−	−	+	−	−	+
	Microcephalus	+	−	+	+	−	+	+	−	+	−	−	−	−	−	−	−	+	−	−	+	−	−	−
	Cachexia/underweight	−	−	−	−	−	+	+	−	+	−	−	−	−	−	−	−	−	−	−	+	−	−	−
	contractures	+	−	−	−	−	−	−	−	+	−	−	−	−	−	−	−	−	−	−	−	−	−	−
	dysmorphia of the face	−	−	−	+	−	+	+	−	+	−	−	−	−	+	−	−	+	−	−	+	−	−	+

*unilateral; Pyramidal tract signs: including spasticity, Babinski sign, autonomic dysfunction: incontinence for urine and bowel. na, not assigned.

#### 2.2.1 ^1^H MRS

For single-voxel spectroscopy (SVS), three T2-weighted MR image sequences, covering the whole brain, were initially acquired in sagittal, axial and coronal orientations, in order to identify the appropriate positioning of the volumes of interest (VOI) within the left-hemispheric hippocampus and the cerebellum. Then, a T1w sagittal volume-rendering magnetization prepared rapid acquisition gradient echo sequence (MP-RAGE, **Table 1**) was acquired. For each hemisphere, a 20 mm×20 mm×20 mm VOI in the hippocampal region and a second VOI in the cerebellum were studied. The hippocampus was chosen since it was reported as one area with most prominent morphological changes in the imaging data by Anttinen et al. [15]. We had to limit the acquisition to one hemisphere due to total MRI acquisition time which prevented us from scanning both sides. We decided to use the left hippocampus as the left hemisphere usually reflects the dominant one. The VOIs for patient and control 2 who were 12 years old was acquired identically even if the inclusion of more parahippocampal tissue was possible since compensation by the pairwise comparison was considered to be appropriate. A first spectrum to check the free induction decay as a marker for shim quality was acquired. All spectra were rejected if the FID did not reach the baseline within 0.3 ms. Proton MR spectra were obtained with a point resolved spectroscopy localization sequence (PRESS) for each VOI (TE 30 ms, TR 2,000 ms, 128 averages, 1,024 data points, delta frequency of −2.7 ppm, bandwidth 1,000 Hz, TA 4 minutes 23 seconds). Reference spectra were acquired for each VOI without water suppression with a long TR of 10,000 ms using two excitations. This spectrum was subsequently used for eddy current correction and for absolute metabolite quantification by fully automatic spectral fitting. The LCModel® software (http://s-provencher.com/pages/lcmodel.shtml) was used to estimate the absolute concentrations of N-acetyl aspartate (NAA), total creatine (Cr) and choline (Cho)-containing substances as well as myo-inositol (mI). LCModel® compares the observed spectrum with a library of spectra from previously acquired model solutions of the respective metabolites.

Due to variations in voxel composition, image segmentation of the MP-RAGE dataset was performed using a fully automated segmentation procedure within the SPM99 software (Wellcome Department of Imaging Neuroscience Group, London, UK; http://www.fil.ion.ucl.ac.uk/spm/software/spm99/), since the MATLAB-based software code of the dedicated ‘spec-vox’ tool box was optimised to SPM99. With respect to individual localization and inclination of the VOIs within the segmented brain, an SPM-based software toolbox using MATLAB® 6.5 (The MathWorks, Natick, MA, USA) was applied to determine the fractional content as a percentage of gray matter (GM), white matter (WM) and cerebrospinal fluid (CSF) within the VOI. The metabolite concentrations were corrected for CSF amounts and partial volume effects, respectively. Here, the metabolite concentrations obtained by LCModel® were divided by the fractional amount of brain tissue. Subsequently, the volume-corrected ratios of NAA divided by Cr, mI, and the sum of Cr and Cho, respectively, and finally the ratio of mI divided by Cr were analysed. The comparison of the values of the metabolite ratios between patients and controls consisted of a Wilcoxon test for non-parametric statistical analysis.

#### 2.2.2 Region-of-interest-based volumetry

Volumetric measurements were applied to the MP-RAGE datasets by use of the interactive software program *MRreg* (Epilepsy Imaging Group, Dept. of Clinical and Experimental Epilepsy, Institute of Neurology, UCL, London, UK; http://www.erg.ion.ucl.ac.uk/MRreg.html) (Lemieux *et al.* 1998). For the complex task of hippocampal tracing, each rater had received an intense training instructed by an experienced rater in data of normal and pathologically altered brains different from the ones used in this study. The manual delineation of the hippocampus was performed according to the protocol, as described by Giesel et al. [16], separately for each hemisphere. Here, the hippocampal measures were initiated three slices rostrally to the point of fusion of the pedunculi cerebri and the pons. Caudally, outlining of the hippocampal formation was terminated at the level where the fornix and the hippocampus fuse. The volume of the hippocampus in each slice (in-slice volume) was calculated by multiplying the voxel number of each trace by the voxel volume and dividing this value by the magnification factor. The total hippocampal volume was calculated as the sum of all in-slice volumes. To obtain the total brain volume, the brain structures were outlined and volumetrically measured in every single slice separately, using the semi-automatic threshold and region-growing tool of the software program *MRreg*. In order to provide a correction for individual brain size, the individual relative hippocampal volume was then calculated by dividing the hippocampal volumes by the total brain volumes. The raters were blind to the subject characteristics. Reliability data was assessed by calculating an intraclass correlation coefficient from repeated measurements [17], [18]. Patient and control groups were compared using the t-test within the Statistical Package for the Social Sciences software (SPSS, Version 12.0, Chicago, IL, USA).

#### 2.2.3 Diffusion Tensor Imaging

DTI scanning protocols are also listed in **Table 1**. Five scans were k-space-averaged online by the scanner software. The complete postprocessing and statistical analysis was performed by use of the DTI analysis software *Tensor Imaging and Fiber Tracking* (TIFT) [19], [20], [21]. First, for the correction of eddy current induced geometric distortions of the echo-planar imaging based DTI data sets, the method proposed by Shen et al. [22] was applied. Second, a spatial normalization routine for all individual DTI data sets as described in Müller et al. [19] was applied. The process requires manually set landmarks according to the Montreal Neurological Institute (MNI) stereotactic standard space [23] performing affine transformation. Fractional anisotropy maps were calculated as previously described [19]. Then, a total of four regions of interest (ROIs) with radius 10 mm (10 analysis voxels, in total 4,169 voxels) were positioned in the thalamus (left and right), the upper corticospinal tract (CST, left and right), the internal capsule (left and right), and the dorsal corpus callosum. The ROI parameter was chosen to be larger than the estimated size of the structure of interest to be secure that the whole structure was covered. Second, since FT is automatically stopped if FA values are <0.2, it was ensured that no low FA regions contributed to the FT and fibre tracts which did not originate from the structure itself could be easily identified and (if not automatically stopped) could be removed. The placement of a ROI in the hippocampus was not possible due to the well-known local eddy current distortion adjacent to the petrous temporal bones. Then, averaged FA values within each ROI were calculated according to [19]. For a comprehensive analysis, fiber tracking (FT) was performed using manually defined startpoints in the thalamus. The thalamic startpoint was chosen since the thalamus was found to be involved in the XP-associated neuropathological process, and hippocampal ROIs could not be chosen due to the low FA values in mesiotemporal areas in DTI data as described above. FT methods were used as described in [21]. In order to quantify the tractography results, the technique named tractwise fractional anisotropy statistics (TFAS) [20] was applied. By this approach, the fiber tracts that originated from an XP-patient data set were used for a statistical comparison to the fiber tracts from the age- and gender-matched control data set. The individual fiber tracts were used for the selection of the voxels that contributed to the following statistical comparison by Student's t-test [20].

## Results

We have conducted a systematic investigation in XP patients to determine the occurrence and characteristics of nervous system involvement. We investigated 14 patients with cytologically determined distinct XP subtypes.

### 3.1 Clinical characteristics

Fourteen patients (6 females, 8 males) were investigated, the mean age at evaluation for the whole group was 22.4±16.2 years, covering a range of 5–68 years). The XP complementation group, the age at evaluation, the age when first XP-specific symptoms were reported, age at onset of XP, the age at manifestation of neurological abnormalities and the body mass indices are summarized in **Table 2**. Most patients included in this study were complementation group XP-C since they represent the largest population of XP patients in Europe. Habitus and neurological findings of all investigated patients are given in **Table 3**, together with what is known to be associated according to the literature. Hallmarks of the phenotype and the individual development are summarised for each patient in the **Text S1**.

In summary, regardless of the XP complemention group and the age at investigation, 10/14 patients (71.4%) showed neurological abnormalities (**Table 3**). Abnormalities of the general habitus consisted of short stature related to the individual age in 5/14, dysmorphia of the face in 4/14, and microcephalus in 2/14 patients. Slowed psychomotor development (childhood milestones, learning disabilities) was observed in 4/14. At investigation, these patients presented with mental retardation and intellectual impairment, but obvious dementia was observed only in one patient. Irrespective of the cognitive status, disinhibition phenomena were observed in five patients. One frequent neurological feature in our patient sample was an atactic syndrome of the lower limbs in 9/14, in five of them with functional deficits on gait function, but ataxia could not be assigned to a clear-cut cerebellar or spinocerebellar syndrome in seven cases while there were three patients with definite cerebellar dysfunction. In three patients, combined pseudobulbar and cerebellar speech disturbances were observed. Definite pyramidal tract signs were observed in 3/14. Other abnormal neurological features included a peripheral neuropathy in two patients, seizures and myoclonus in at least one patient.

### 3.2 Conventional MRI data

**Figure 1:**
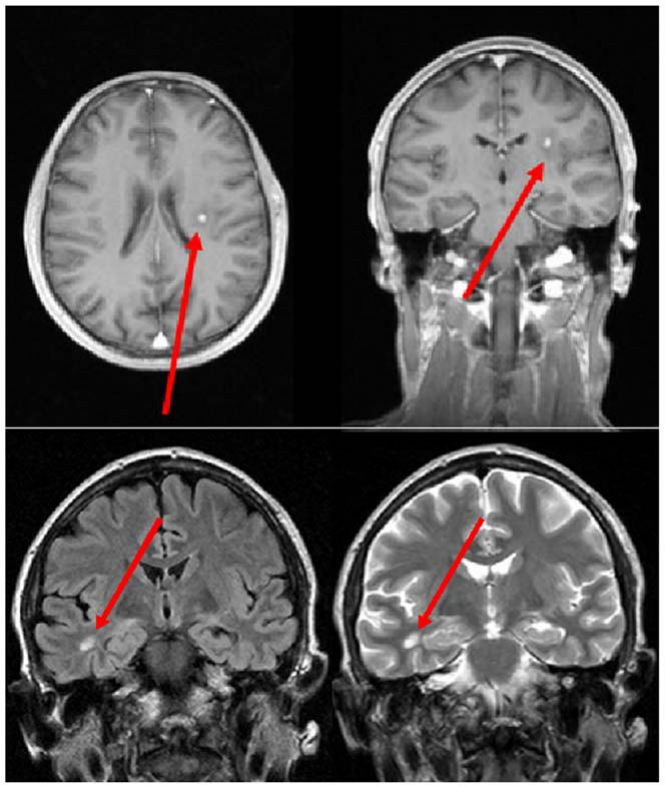
Case 8 (XP-C). Upper panel: T1-weighted conventional MRI post contrast in axial and coronal view. The arrow highlights a left-hemispheric contrast-enhanced lesion with perifocal edema within the frontoparietal white matter. Lower panel: Additional lesion in the temporal white matter presenting as a hyperintensity in coronal FLAIR (left) and T2-weighted (right) scans, marked by arrow. (For details, please cf. 3.2 Conventional MRI data.).

The results of the evaluation of the conventional MRI data are summarized in the **Text S1**. Only one finding is to be described in detail: in Case 8 (XP-C), MRI showed multiple spatially dissemininated hyperintense white matter lesions, some of which presented with a perifocal edema, some also showed a marked homogeneous enhancement of contrast medium, one lesion additionally demonstrated an abnormal signal in DWI. In summary, the imaging findings of the bihemispheric lesions with dissemination in time were compatible with an inflammatory process (**Figure 1**).

### 3.3 Multiparametric MRI data

#### 3.3.1 ^1^H MRS

Compared with each matched control, the NAA/Cr ratios and the NAA/(Cr+Cho) ratios were lower in 5 out of 7 patients in the hippocampal VOI (exceptions, cases 1 and 2). The NAA/mI ratios were lower in all patients, while the mI/Cr ratios were higher in all patients except from case 13. In the cerebellar VOI, the NAA/Cr ratios, the NAA/(Cr+Cho) ratios, the NAA/mI ratios, and the mI/Cr ratios did not show a consistent pattern of results, since the patients showed lower or higher values in 3 out of 6 matched pair analyses each, in various combinations.

**Figure 2:**
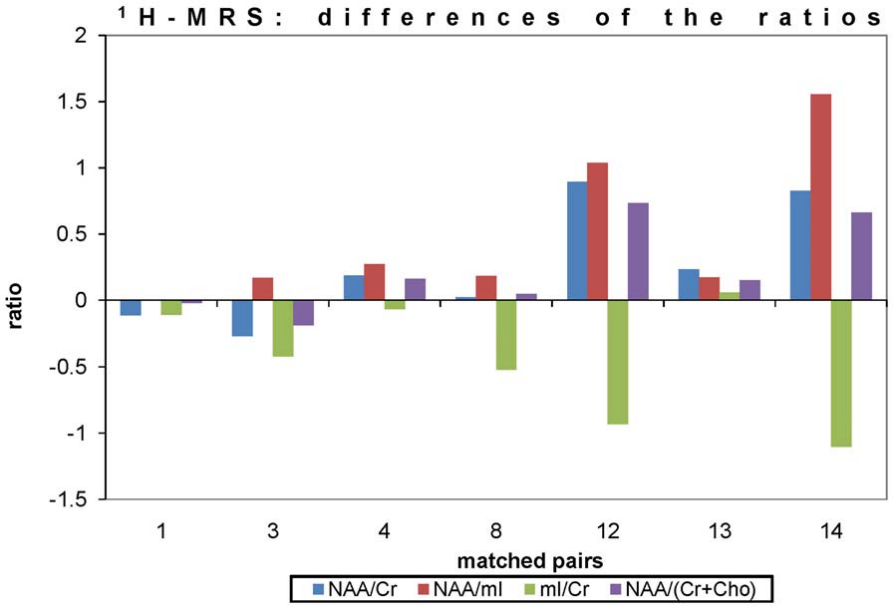
Results of ^1^H MRS analysis: Differences of the metabolite ratios for each age- and gender-matched pair (cases 1, 3–7 and 9). The given values are the ratios of the patients subtracted from those of the controls. Different metabolite ratios are marked by distinct symbols, the rhomb (dark blue) refers to NAA/Cr, the square (turquoise) refers to NAA/mI, the triangle (orange) refers to mI/Cr, and the point (green) refers to NAA/(Cr+Cho), respectively. [NAA = N-acetylaspartate; Cr = creatine; mI = myo-inositol; Cho = choline].

The statistical comparison of the volume-corrected metabolite ratios, i.e. NAA/Cr, NAA/mI, NAA/(Cr+Cho), and mI/Cr, by the Wilcoxon test showed significant differences between patients and controls for the hippocampal VOI, whereas no statistical differences could be observed in the cerebellar VOI. The results are summarized in **Table 4**. The values of the four ratios in the hippocampal VOI as differences between patients and controls are demonstrated in **Figure 2**.

**Table 4:** Level of significance of the Wilcoxon-test of the volume-corrected metabolite ratios derived from the hippocampal and the cerebellar volume-of-interest (VOI), respectively.

VOI	Metabolite ratios (volume-corrected)			
	NAA/Cr	NAA/mI	NAA/(Cr+Cho)	mI/Cr
Hippocampus	<0.05	<0.01	<0.05	<0.05
Cerebellum	n.s.	n.s.	n.s.	n.s.

NAA, N-acetylaspartate; Cr, creatine; mI, myo-inositol; Cho, choline. n.s., not significant.

#### 3.3.2 Volumetric measurements

**Figure 3:**
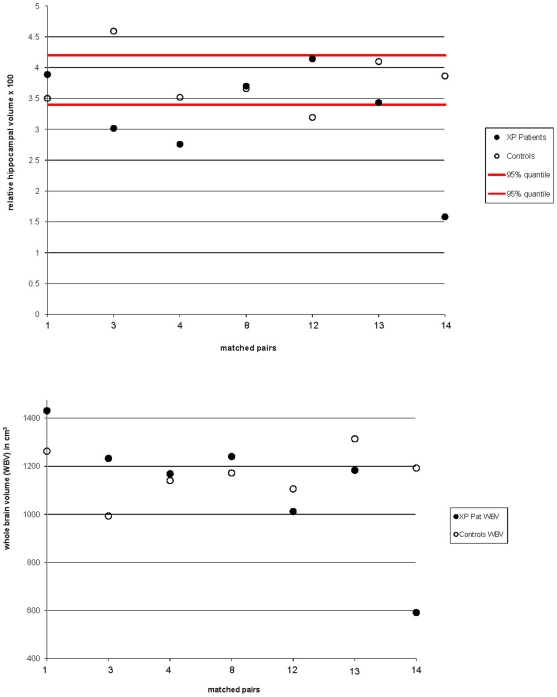
Results of the volumetric measurements: upper panel, relative bilateral hippocampal volumes for pairs of XP patients and corresponding controls; lower panel, whole brain volumes (WBV) for pairs of XP patients and corresponding controls. The 95% quantile of the controls' hippocampal volumes are indicated as red lines.

**Table 5** summarizes the volumetric findings. Neither a reduced global brain volume nor regional hippocampal atrophy was observed at group level. The absolute values for hippocampal volumes ranged between 3.22 and 5.57 cm^3^ in the patient sample with one exception (patient 14, aged 23 years, 0.94 cm^3^), while the hippocampal absolute volumes in the age-matched pairs control sample ranged between 3.53 and 5.39 cm^3^ (compare **Table 5**). For the individual normalized volumes of the matched pairs, please see **Figure 3**. Both for left and for right hippocampus, the patients' mean volumes were lower than the controls', with no significant asymmetry effect (left vs. right hippocampal volumes, p = 0.581 in patients and p = 0.571 in controls). The whole brain volumes (WBV) were 1,122.7±256.1 cm^3^ in patients and 1,168.6±105.0 cm^3^ in controls (p = 0.677). The total hippocampal volumes, both as the absolute value and corrected for brain size, were lower in the patients. This finding has to be judged as a trend since it failed to reach statistical significance (p = 0.159 for relative volumes). Examination at the single age-matched pair level showed that in all patients with a relative hippocampal volume below the 95% quartile of the controls (3.349) (pairs 3, 4 and 14; **Figure 3**, upper panel) had clearly smaller relative hippocampal volumes than their corresponding controls. Patients with relative hippocampal volumes within the 95% quantiles of the controls showed no reduced relative volumes in 3 of 4 cases compared to the controls (pairs 1, 10, 11 and 13 in **Figure 3**, upper panel). Except from pair 14, the corresponding matched pair whole brain volumes differed only marginally (**Figure 3**, lower panel).

**Table 5:** Results of volumetric analysis in XP patients and controls.

	proportion of right hippocampal to whole brain volume[×10^−3^]	proportion of left hippocampal to whole brain volume[×10^−3^]	total hippocampal volume[cm^3^]	proportion of hippocampal to whole brain volume[×10^−3^]
XP patients	1.601±0.329	1.616±0.546	3.755±1.443	3.217±0.867
controls	1.844±0.219	1.930±0.256	4.400±0.571	3.775±0.460
t-test	P = 0.130	P = 0.194	P = 0.239	P = 0.159

#### 3.3.3 Diffusion Tensor Imaging

**Figure 4:**
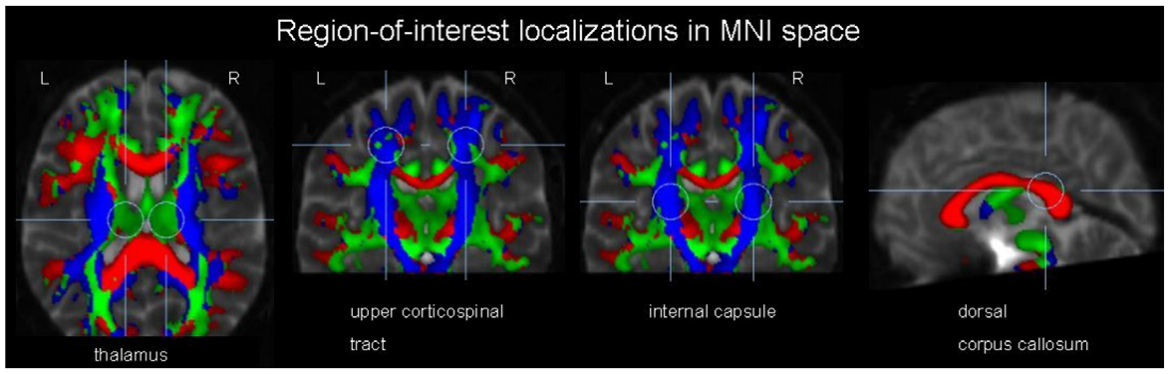
Localization of the regions-of-interest (ROIs) for FA analysis of the DTI data, overlain onto a b = 0 data set together with a colour-coded FA-map (red, right-left direction; green, anterior-posterior direction; blue, craniocaudal direction). From left to right, the bilateral thalamic ROIs are presented in an axial slice, the bilateral ROIs in the upper corticospinal tracts in a coronal slice, the bilateral ROIs in the internal capsule in a coronal slice, and the ROI in the dorsal corpus callosum in a sagittal slice. MNI, Montreal Neurological Institute standard space.

In **Table 6**, the DTI results (mean values of the groups patients and controls, respectively) are presented for the four different ROIs (which are demonstrated in **Figure 4**) with respect to number of voxels and average FA. The voxels with FA-values>0.2, i.e. the number of voxels with a high degree of directionality, was significantly lower in the XP patients in all ROIs, with the most marked differences in the thalamus and the internal capsule. The average FA was also significantly lower in the patients, thus demonstrating a reduced level of directedness within the ROIs. (The differences between ROIs are due to the physiological neuroanatomical variability of directionality in different areas of the brain.) In summary, all selected areas were found to exhibit white matter fiber changes in association with XP.

**Table 6:** Results of the region-of-interest (ROI)-based analyses of five patients and five age- and gender-matched controls.

ROI	MNI coordinates *x/y/z*	number of voxels with FA>0.2	average FA	significance level
thalamus patients	(−)11/−21/18	2426±662	0.22±0.04	voxel number: p<0.01average FA: p<0.05
thalamus controls		3113±169	0.26±0.01	
upper corticospinal tract patients	(−)22/−21/47	3583±447	0.36±0.07	voxel number: p<0.05average FA: p<0.05
upper corticospinal tract controls		3915±171	0.43±0.04	
internal capsule patients	(−)23/−21/15	3347±307	0.37±0.04	voxel number: p<0.001average FA: p<0.001
internal capsule controls		3816±190	0.45±0.03	
corpus callosum patients	0/−35/20	2377±519	0.29±0.12	voxel number: p<0.05average FA: p<0.05
corpus callosum controls		3237±239	0.44±0.06	

FA values are given as mean ± SD. Significance level was determined by Student's t-test. Left- and right-hemispheric ROIs are arithmetically averaged for each localisation (except from corpus callosum where only one ROI was selected) since there were no asymmetric findings in all of them. MNI, Montreal Neurological Institute; FA, fractional anisotropy.

**Figure 5:**
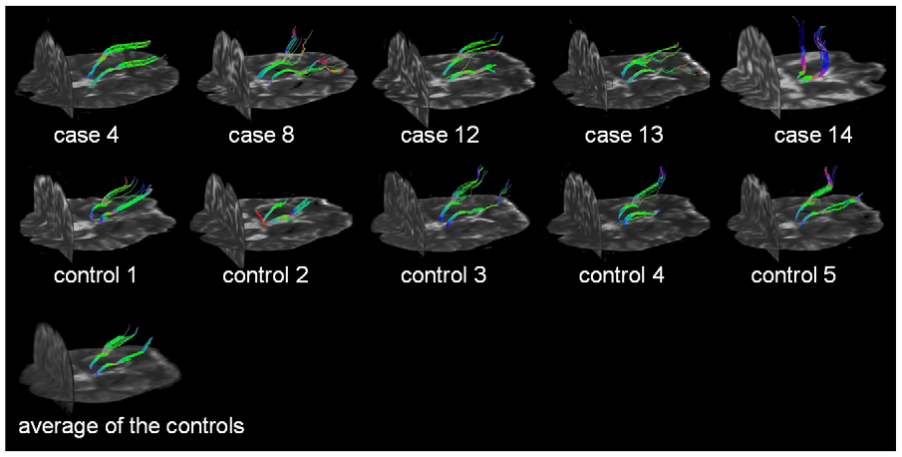
DTI-based fiber tracking (FT) with starting points in the thalamus for the individual XP patients (upper panel) and the age- and gender-matched controls (center panel). FT was additionally performed in an averaged data set obtained from the data sets of the five controls (lower panel). Background is the individual b = 0 data set.

**Figure 5** shows the FT results with starting points in the thalamic region (as the area with the most prominent differences) pairwise for XP patients and each corresponding control. The tract projections mainly show a posterior to anterior direction. In some patients, depending on severity of the disease, the analysis showed a different tract behaviour in the thalamic region which may be caused by a low signal to noise ratio when the directional information gets lost. The quantitative TFAS analysis resulted in significant differences (p<0.005) between the FA values of the voxels determined by FT in patients and in controls for all the age- and gender-matched pairs (**Table 7**).

**Table 7:** Results of the TFAS analysis of five patients and five age- and gender-matched controls.

	number of voxels in the fiber tracts	average FA	significance level
patient no. 4	2831	0.32±0.13	p<0.001
control no. 1	2556	0.35±0.13	
patient no. 8	2590	0.30±0.13	p<0.001
control no. 2	2075	0.33±0.12	
patient no. 12	1496	0.26±0.10	p<0.001
control no. 3	2046	0.30±0.11	
patient no. 13	1838	0.26±0.09	p<0.001
control no. 4	2192	0.30±0.11	
patient no. 14	1868	0.28±0.10	p<0.005
control no. 5	1764	0.29±0.11	

FA values are given as mean ± SD. Significance level was determined by Student's t-test. FA, fractional anisotropy.

## Discussion

### 4.1 Summary

XP is the first progeroid syndrome in which defective DNA damage repair mechanisms account for neurodegeneration and early neural developmental disabilities [8]. In the past three decades, neurological abnormalities involving both the central and peripheral nervous system and including low intelligence, spasticity, ataxia, areflexia and hearing loss were observed in the XP complementation groups A, B, C, D, F and G [2], [5], [14], [24], [25]. Japanese XPA patients showed neurological abnormalities more often than patients belonging to other XP complementation groups [14]. The more severe the molecular XP defect is, the more pronounced the severity of the neurological phenotype seems to be, opening an approach for a prognostic assessment of XP patients [26].

In a recent study on the natural course of XP in 16 Finnish patients, Anttinen and colleagues [15] reported that the neurological symptoms probably start slowly at about the age of 2 and manifest at the age of 4–5 years as cognitive and cerebellar signs, then slowly progress and finally affect the whole nervous system. However, since the cause of the neurological disease is still unknown (it cannot be caused by the UV photoproducts because UV radiation penetrates only into the skin), our understanding of the origin of the XP-associated neurological abnormalities is still in its infancy so that Anttinen *et al.* conclude that neurological problems in XP should be the focus of future research [15]. Beyond pure morphological information on brain structures *grosso modo* as available from conventional MRI or (even to a much lesser degree) from CT, advanced MRI-based neuroimaging techniques are able to provide quantitative macrostructural and even microstructural assessment of the brain *in vivo*.

### 4.2 Clinical data

The spectrum of the clinical neurological abnormalities in the complete cohort was – although in general agreement with it – beyond the XP patients described in the literature: the most common features were a mild to moderate ataxia, pronounced in the lower limbs (N = 8) and clinical signs or symptoms of pyramidal tract involvement in terms of spasticity/spastic gait, preventing only two patients from walking without assistance. Other common neurological symptoms were unspecific cognitive deficits and a reduced psychomotor development (N = 4) and abnormal behaviour (N = 5). Four of the patients had not reached the age of ten years so that an early affection of the CNS, including the cerebellar system and the corticospinal tracts, could be demonstrated in the cross-sectional design. None of these young XP patients was able to take part in the neuroimaging study due to fear and motor restlessness. From the clinical viewpoint, in most of these patients a clear classification to a distinct cerebellar or spinocerebellar syndrome was difficult. In the total group, a definite cerebellar syndrome including dysmetria and intention tremor were observed in two cases with XP-A and TTD/XP-D. Dysarthrophonia in a total of four patients was clinically a mixture of cerebellar and pseudobulbar speech in three patients. Pseudobulbar speech, as a feature of pyramidal tract dysfunction, was present in all patients with definite limb signs of pyramidal tract abnormalities.

Most patients in our study were from complementation group XP-C due to highest prevalence of this group in Europe. These patients did show neurological symptoms (**Table 3**) correlating with morphological analysis. This is in contrast to the study by Anttinen and colleagues [15] where no neurological symptoms were found in XP-C patients, perhaps due to young age. However, also in their study, mild morphological changes were detected by MRI and the authors hypothesize that oxidative damage may be the reason for neurological changes. From our point of view, this may be a possible explanation even though involvement of the respective proteins has not been shown for all XP proteins. Investigation of clinico-morphological correlations such as studies by Anttinen et al. and us will provide the possibility to systematically investigate the role of different repair proteins in the development of neurodegenerative symptoms.

There was one young patient with XP-D/trichothiodystrophy (TTD) overlap syndrome; due to different disease causing mutations in the DNA repair genes XP-D and TTD, the genetic background of the autosomal recessive TTD is heterogeneous [27]. It is well-known that TTD patients *per se* show a heterogeneous phenotype, including developmental and neurological abnormalities (besides cutaneous and endocrinological symptoms), and the patient from our cohort showed severe neurological deficits with cerebellar symptoms, spastic tetraparesis and mental impairment, progressively starting at an age of 18 months. The XP-D gene product is required for nucleotide excision repair and is one of the components of basal transcription factor TFIIH as well. Therefore, different mutations in the XP-D gene may result in a variety of clinical manifestations, from pure mild XP disease to a severe phenotype with premature and infantile developmental disabilities and progressive neurological deterioration [28].

Few Caucasian patients from the complementation group XP-F are described in the literature since these are mostly of Japanese origin. Most of them have exclusively dermatological abnormalities so that XP-F was considered a mild form with late onset [29], although there is one report about a young boy with XP-F and a severe progeroid syndrome including mental retardation and ataxia [2]. The patient of our group, in her late sixties, showed only a mild axonal sensorimotor neuropathy with consequent afferent ataxia. Regarding the estimated frequency of polyneuropathies between 25 to 50/100000, it remains unknown whether the neuropathy in our patient was XP related [30], [31] although the common causes for polyneuropathy were excluded. Up to now, the only XP groups associated with neuropathy were XP-A and XP-G in which mixed axonal-demyelinating neuropathies were described [10], [12], [32].

### 4.3 Neuroimaging data

Seven patients with XP (three XP-C, one XP-A, one XP-F, one XP-V, one unassigned case) out of the 14 patients cohort received multiparametric MRI in order to assess the cerebral morphology *in vivo* at a macrostructural and microstructural level in comparison to controls. We aimed to analyze both grey matter structures with respect to volumes and metabolism by volumetric and ^1^H MRS measures, respectively, and white matter microstructural organisation by DTI. The pattern both for GM and for WM alterations was heterogeneous among the group. Given that Anttinen et al. also described a variation of neuroimaging results in patients with XP complementation groups A and C [15], our cohort which consisted of even more complementation groups allowed for even more clinico-morphological variation. For future studies, these results may be of interest with respect to other diseases of the central nervous system, in particular neurodegenerative diseases such as the dementias. Neurological symptoms and neuroimaging may be aligned with those findings in XP (where the genetic and molecular defects are known), possibly giving indications which clinical symptom may be caused by defects in DNA repair or oxidative stress and which not.

It could be shown that single patients show a marked hippocampal and whole brain atrophy but most of the patients investigated in this study were not different from controls at this quantitatively assessed macroscopic level. However, it could be demonstrated that the hippocampal formation, although not prominently thinned, was metabolically altered with lowered NAA-based ratios as a marker of neuronal integrity and increased MI-based ratios which is used both as a glial marker and particularly a product of the degradation of myelin. Remarkably, the cerebellar VOI did not demonstrate metabolic changes neither at group nor at subject level, although clinical cerebellar signs were present in single patients. It might be concluded that the process of regional degeneration initiates in the hippocampal formation before the cerebellum. For that purpose, longitudinal ^1^H MRS data would be necessary. Other regions could not be assessed with respect to metabolism, since the analysis had to be restricted to VOIs due to technical prerequisites and the bilateral hippocampus and the cerebellum were chosen as they were previously reported as areas involved in the XP-related CNS affection.

The analysis of WM microstructure by use of DTI brought the most striking results. The thalamus, the corticospinal tracts and the dorsal corpus callosum were selected *post acquisition* for white matter directionality analysis since pyramidal tract pathology seemed to be obvious from the clinical phenotype. Unfortunately, hippocampal and cerebellar WM tracts could not be assessed due to the well-known local interferences in DTI data adjacent to the petrous temporal bones and the limitations of the field of view. The WM directionality was reduced in all areas in the XP patients, irrespective of the complementation group. Of whatever etiology the WM process is, it seems to be a generalised one since all WM areas investigated were shown to be markedly altered with thinning of fibertracts and no correlation with the clinical presentation could be observed (e.g., pyramidal tract abnormalities were not associated with pyramidal signs). Although DTI does not differentiate between antero- and retrograde degeneration processes, a primary WM process in association with XP is most probable, given the lack of whole brain atrophy corresponding to cortex structures. In addition, a finding in conventional MRI in one XP-C patient was most remarkable, with multiple spatially disseminated hyperintense lesions, partly with perifocal edema and a marked homogeneous enhancement of contrast medium (**Figure 1**), compatible with an inflammatory process with dissemination in time and reminiscent of MRI findings in multiple sclerosis. These lesions fulfilling imaging criteria of acute inflammation have to be considered a by chance finding. It has been reported that patients with XP show immunosuppression in cellular immunity. Cells from XP patients can show abnormally high rates of apoptosis after cellular stress such as ultraviolet radiation [33]. It is tempting to speculate that high levels of oxidative stress, i.e. cyclopurines [34], may induce apoptosis in neurons which in turn can lead to cerebral inflammatory reactions, similar to autoimmune disease, as observed in this patient. However, this is speculative and this question can only be investigated when neuronal cells from patients with DNA repair deficiency syndromes are available. It might be worthwhile to perform MRI scanning on a regular basis in XP to screen for potential pathological processes and to correlate it with cerebrospinal fluid analysis. This finding is in favour of the latter position within the current discussion whether the myelin structures did form insufficiently during development or myelin structures degrade prematurely in the brain in these patients [26].

Some limitations of our study need to be addressed. First, the study sample is small and heterogeneous, due to the rareness of XP. Methodologically, we thus were cautious in the interpretation of the effects and limited most analyses to a comparison of matched pairs. Second, there was a bias in that the more severe affected patients, in particular with respect to cognitive and neuropsychological abnormalities, could not be investigated by MRI due to non-compliance and motion artifacts so that the results observed in this study might be considered as even less pronounced than they might be found in the clinically more severely affected patients. As one example, patient 14 in this study who presented with the most severe neurological phenotype of those patients who received MRI showed the most prominent results in the imaging analysis. Other limitations are caused by methodological restrictions of the neuroimaging data acquisition: for a complete multiparametric analysis of the ROIs, an SVS analysis of the WM of the motor system including CST or the corpus callosum would have been useful as well as a DTI-based analysis of the hippocampi. The first could not be included since a specific VOI placement was not done due to a different a priori hypothesis, the latter could not be performed due to the well-known local eddy current distortion adjacent to the petrous temporal bones.

### 4.4 Conclusion

The primary defect in XP is the response to DNA damage. Given that aging results from accumulated damage [2], that each tissue has different requirements for the various repair mechanism [35], and further that since reactive oxygen species play a central role in generating aging-associated DNA damage, it appears possible that endogenous damage caused by reactive oxygen species, a consequence of the high rate of oxidative metabolism in the brain, is the underlying cause of neuropathogenic DNA lesions. DNA lesions induced by reactive oxygen species have the potential to block transcription by RNA polymerase II, and their accumulation in repair-defective individuals may cause neuronal cell death, e.g. through apoptosis [36]. Many neurodegenerative diseases caused by molecular defects in DNA repair are clinically either ataxias or motor neuron degeneration followed by cognitive decline and impairment of neurological systems. The correlation between molecular defect and neuron-specific impact may provide the missing link that allows us to begin to understand the relationship between neurodegeneration and defective DNA repair [9], [37]. With regard to the present study, a vulnerability pattern of this neurodegenerative process could be assessed *in vivo* in the brains of XP patients which was observed across complementation groups. The prominent neuroanatomical correlates seem to be WM alterations involving both craniocaudal fibers such as the pyramidal tracts and interhemispheric connections such as the corpus callosum. Thus, WM affection could be demonstrated to be prominent in association with XP, in contrast to several reports of prominent GM alterations. In addition, the hippocampus is a structure which could be shown to be reduced in volume in single patients and to be pathologically altered in metabolism at group level. In contrast, whole brain atrophy was not a feature of XP in our sample, and cerebellar involvement – although obvious in the clinical domain – could not be related to neuroimaging findings.

## Supporting Information

Text S1 Conventional MRI data.
(DOC)
