# Supplementary material for: The Cerebro-Morphological Fingerprint of a Progeroid Syndrome: White Matter Changes Correlate with Neurological Symptoms in Xeroderma Pigmentosum
Source: PLoS One. 2012 Feb 21;7(2):e30926. doi: 10.1371/journal.pone.0030926 (PMC3283603; doi:10.1371/journal.pone.0030926)
Supplement: Text S1 — Conventional MRI data. (DOC) [file pone.0030926.s001.doc]

**Conventional MRI data**

Case 1 (XP-A)

The lateral ventricular spaces were symmetrically enlarged, in particular with respect to the temporal and dorsal horns, possibly as an *e vacuo* increase in size. Cortical areas showed no atrophy, including the hippocampi, although both hippocampi and amygdala had irregular signal intensities. A single subcortical lesion was found in the left-hemispheric frontal white matter.

Case 3 (XP-C)

Subtle signal inhomogeneities in the bilateral hippocampus formations and a single right-hemispheric subcortical lesion close to the posterior horn of the lateral ventricle (in T2w and FLAIR, presenting as a black hole in T1w) were observed. Otherwise no pathologies were found, in particular no cortical atrophy or additional focal lesions.

Case 4 (XP-C)

The bilateral hippocampus showed subtle signal hyperintensity (FLAIR), but no atrophy. The dorsal parts of the corpus callosum were thinner than usual. Multiple supratentorial subcortical lesions both in periventricular and in juxtacortical localisations were observed, in addition an irregular lesion of uncertain etiology/dignity in the area of the right dorso-medial thalamus with a diameter of about 10 mm (T2w, FLAIR).

Case 6 (XP-C)

No pathological MRI findings were observed, in particular no global or regional atrophy and no circumscribed lesions.

Case 8 (XP-C)

No global or locoregional atrophy was observed, including the normal-appearing structures of the hippocampi and the cerebellum. In contrast, the white matter showed multiple spatially dissemininated hyperintense lesions (T2w, FLAIR), some of which presenting with a perifocal edema, most prominent in a right-sided temporal and a left-sided frontoparietal lesion. These two lesions also showed a marked homogeneous enhancement of contrast medium, the left-hemispheric lesion additionally demonstrated an abnormal signal in DWI. In summary, the imaging findings of the bihemispheric lesions with dissemination in time were compatible with an inflammatory process (**Figure 1**).

Case 12 (XP-F)

Both the bilateral hippocampus formations and the bilateral putamen and the thalamus presented with diffuse inhomogeneous hyperintensities (T2w, FLAIR). T2w and FLAIR also demonstrated multiple subcortical white matter lesions in periventricular and in juxtacortical localisations. There was no cortical atrophy.

Case 13 (XP-V)

No pathological MRI findings were observed, in particular no global or regional atrophy and no circumscribed lesions.

Case 14 (unassigned)

A marked supratentorial cerebral atrophy was observed, including an *e vacuo* enlargement of all ventricular spaces and an additionally prominent focal atrophy of the bilateral hippocampal formations together with pronounced hyperintensities of the hippocampi. In most parts of the subcortical white matter, signal alterations as diffuse hyperintensities (T2w, FLAIR). The corpus callosum was found to be markedly thinned in all of its parts. Also the infratentorial structures showed a prominent volume reduction of cerebellar parts (inferior portion and vermis) and the cerebellar pedunculi, whereas the cerebellar hemispheres were relatively preserved.
